# Developing and testing a system alignment approach to address homelessness among black fathers in Atlanta, GA

**DOI:** 10.1186/s12889-026-26791-w

**Published:** 2026-02-27

**Authors:** Latrice Rollins, Thomas Cotton, Lease Youmans, J. Dontae Roberts, Jennifer Elliott, Diamond Cunningham, Caleb Platel, Chris White, Jasmine Cunningham, Errol Crook

**Affiliations:** 1 Morehouse School of Medicine, Atlanta, GA USA; 2 Redemption & Advancement Alliance, Inc, Atlanta, GA USA; 3 North Carolina Agricultural and Technical State University and Black Legacy Alliance, Greensboro, NC USA; 4 University at Buffalo, Buffalo State University, Buffalo, NY USA; 5 Tulane University, New Orleans, LA USA; 6 Family Outreach Ministries Int. Inc, Atlanta, GA USA; 7 Father Movement, Stockbridge, GA USA; 8 Partners for Home, Atlanta, GA USA

**Keywords:** Fathers, Homelessness, System alignment, Community-based participatory research, Functional zero

## Abstract

**Background:**

Homelessness is a profound public health crisis in the United States, elevating risks for chronic illness, mental health disorders, substance use, and family instability. Homelessness response systems remain structurally centered on maternal caregiving, routinely barring adult men, including fathers, from accessing family shelters. Functional Zero is a structural intervention that supports dynamic tracking of individuals through a by-name list and seeks to reduce the number of individuals experiencing homelessness to zero in a community. The purpose of this study was to assess Functional Zero as a systems alignment strategy to address homelessness among Black fathers in Atlanta, GA.

**Methods:**

Using a community-based participatory approach and the Exploration, Preparation, Implementation, and Sustainment (EPIS) framework, we developed and tested a Functional Zero model with fathers, medical, social service, and public health partners from February 2023 to May 2024. We conducted interviews with these partners (n = 8) and fathers (n = 6). Rapid qualitative analysis was used to identify themes. We reviewed data from Atlanta’s Homelessness Management Information System to assess data collected about fathers and prevalence. A survey completed by coalition members (n = 8) assessed satisfaction, impact, and sustainability of a Functional Zero approach.

**Results:**

Most fathers experiencing homelessness in Atlanta identify as Black (93%) and single (53%). Partners reported a lack of data about fathers, a limited focus on serving and documenting fathers, and the need for a coordinated system with dedicated staff. Fathers described chronic housing needs and barriers to accessing resources.

**Conclusions:**

Functional Zero requires transforming how systems identify and support father-led families, shifting from shelter management to prevention and rapid resolution. Policies should expand family and homelessness definitions, require father inclusion in services, and invest in cross-sector data systems tracking intersectional populations.

**Supplementary Information:**

The online version contains supplementary material available at 10.1186/s12889-026-26791-w.

## Introduction

Homelessness is a profound public health crisis in the United States, elevating risks for chronic illness, mental health disorders, substance use, and family instability [1, 2]. Black families experience homelessness at disproportionately high rates, yet few programs are designed to meet the unique needs of this population [3, 4]. Although Black individuals comprise 13% of the U.S. population, they account for nearly 40% of people experiencing homelessness, an overrepresentation rooted in structural racism, housing discrimination, and economic marginalization [4, 5]. In Atlanta, where 80% of the homeless population is Black and the majority are men, the local crisis mirrors and intensifies national patterns of gendered and racialized housing exclusion [6]. About 41% of men experiencing homelessness are fathers, yet only 16% of sheltered families include a father, reflecting systemic exclusion from family housing programs [4, 7]. Homelessness response systems remain structurally centered on maternal caregiving, routinely barring adult men, including fathers, from accessing family shelters [8] This exclusion intensifies for Black fathers, who encounter compounded barriers driven by systemic racism, economic instability, and involvement with punitive social systems [9] These barriers erode trust and limit access to coordinated care [4, 8]. Black fathers experiencing homelessness report profound insecurity in their parenting roles, frequently hindered by shelter policies, stigma, and a lack of supportive social services and networks [4].

Despite these inequities, few studies have examined homelessness through an intersectional lens that accounts for race, gender, and parental status [10–14]. Most research aggregates data in ways that obscure the specific experiences of Black fathers, and even fewer studies have tested system-level interventions to address their unique housing needs [15] As a result, homelessness response systems remain inequitable by design and in implementation with Black fathers [16].

Functional Zero is a framework designed to end homelessness by ensuring that housing placements consistently outpace new inflow. It emphasizes real-time data, shared accountability, and cross-sector coordination to manage homelessness at the population level [17]. More than a programmatic fix, Functional Zero is a structural intervention that supports dynamic tracking of individuals through a by-name list and fosters collaboration between housing, health, and social service systems [18]. Communities across the United States have used Functional Zero to reduce veteran and chronic homelessness, yet the model has not been evaluated with a racial equity lens or tailored to meet the specific needs of Black fathers [18, 19]. Applying Functional Zero in this context offers an opportunity to assess its adaptability for historically excluded populations and examine how systems can be reoriented to close racial and gender-based service gaps. Guided by a theory of change grounded in inclusion, coordination, and structural accountability, Functional Zero represents a promising model for transformative systems reform.

This study, Research to Understand Systems for Housing (RUSH), was a pilot project that brought together partners from public health, medical, and social services in Atlanta, Georgia, using a community-based participatory research (CBPR) approach from February 2023 to May 2024. The two main goals of the project were to identify barriers and facilitators within the homelessness response system for medical, public health, and social service providers serving fathers, and to assess the feasibility of implementing Functional Zero to address fathers’ housing challenges.

## Methods

The RUSH project employed community-based participatory research (CBPR) approaches and the Exploration, Preparation, Implementation, Sustainment (EPIS) framework to inform the inclusion of father-focused homelessness services across various systems. Together, these approaches provided a guiding structure for both engaging partners and systematically testing the Functional Zero approach, as described below.

### Community-based participatory research

A key aspect of CBPR is that the prioritized communities are involved in all stages of the research process [20, 21]. This involvement eases the translation of research to practice, a common barrier in more traditional research processes [20, 22]. CBPR methods utilizing high levels of community involvement in the planning process are more likely to result in high levels of benefit, trust, and satisfaction within the population being served, which, in turn, broadens the scope and sustainability of the intervention [20, 21]. A key factor in introducing successful programs and interventions to address persistent societal issues is engaging the affected community from the outset and maintaining ongoing engagement throughout the planning, development, and implementation of selected interventions [20, 21].

The RUSH Project Coalition (RPC) was established at the beginning of the study to optimize the alignment between RUSH and a range of homeless services across diverse contexts. The RPC included leaders from the medical, public health, and social service organizations that serve fathers experiencing homelessness. The RPC also included fathers with lived experience of homelessness, incorporating both current, lived experience and lived experience of recovery from a homelessness crisis (where possible). The RPC met bi-monthly to guide study design choices, interpret results, implement findings in diverse settings, and evaluate the collaborative process.

### Exploration, preparation, implementation, and sustainment (EPIS) framework

The EPIS framework was used to assess intervention feasibility because it systematically examines factors from the initial idea (Exploration) through readiness (Preparation) to Implementation and long-term continuation (Sustainment), analyzing both internal (organizational) and external (contextual) barriers and facilitators [23]. The following sections will describe the study methods and findings in relation to the EPIS framework.

### Secondary data analysis

The Exploration phase of the EPIS framework helps to assess the need for the intervention. To understand the landscape of homelessness among fathers in Atlanta, we requested and reviewed existing aggregate, de-identified client-level data from the Atlanta Homeless Management Information System (HMIS). The most recent demographic and service data were requested for the households in the HMIS by race and relationship status (single or partnered) for males. All data were pulled using the HMIS Active Client List custom report, deduplicated, and sorted by the most recent service type enrollment date.

### Partner interviews

In the Exploration phase of EPIS, the needs and barriers in systems serving fathers experiencing homelessness must also be identified. To better implement the Functional Zero approach and understand the current context of each partner organization, we conducted eight qualitative interviews with our medical, social service, and public health partners (See Supplementary File for Interview Guide). The interviews aimed to identify strengths and gaps in our local homeless response system, define its operation and flow, and explore factors affecting fathers’ outcomes. Interviews lasted 30 to 60 min and were conducted by the research team. Interview audio was recorded and transcribed verbatim.

Rapid qualitative data analysis was used to facilitate the timely synthesis of interview data and support feedback from the RPC. Interview transcripts were summarized using a structured analytic template aligned with the study aims and a priori domains of interest based on the EPIS framework. This approach allowed two members of the research team to efficiently identify salient themes, patterns, and points of convergence and divergence across participants [24, 25]. Preliminary findings were synthesized across interviews and presented to the RPC for collective interpretation, contextualization, and validation, consistent with participatory analytic practices that enhance rigor, relevance, and credibility [26, 27].

#### Father participant criteria

During the Preparation phase of the EPIS framework, systems must ready themselves to implement change. Based on partner interviews and RPC meetings, we learned that the definition of homelessness can be a barrier to services. We decided to broaden our definition of homelessness, modifying the one used in the Workforce Innovation and Opportunity Act (WIOA) [28]. The RPC collaboratively agreed that we would use the following definition of homelessness to identify fathers:

- An individual or family who lacks a fixed, regular, and adequate residence.
- An individual or family with a primary residence that is a public or private place not designed for or ordinarily used as a regular sleeping accommodation for human beings, including places like cars, parks, abandoned buildings, bus or train stations, airports, or camping grounds.
- An individual or family living in a supervised, publicly or privately operated shelter designated to provide temporary living arrangements, which includes hotels and motels paid for by government programs or charitable organizations.
- An individual who resides in a shelter or place not meant for human habitation and is exiting an institution where they temporarily resided.

We then decided to focus our pilot study on fathers who:

- Identify as Black/African American.
- Are 18 years of age or older.
- Are custodial fathers (children w/father; father w/partner & children).
- Live in the city of Atlanta.
- Meet the definition of experiencing homelessness.

### By-name list

In the Implementation phase of the EPIS framework, social services and public health systems must move from planning to action. Additionally, the Sustainment phase involves institutionalizing these practices for long-term public health impact. Communities that have sought to end homelessness emphasize the importance of implementing a by-name list (BNL), a comprehensive list of people experiencing homelessness, in addition to the comprehensive program data contained in a community’s HMIS. A well-maintained BNL, updated in real-time for target populations, helps homelessness response systems in two ways: first, by coordinating care at the individual level, and second, by displaying population-level trends, allowing partners to problem-solve and providing them with a feedback loop on progress. Information about system inflow (people entering homelessness) and outflow (people exiting the homelessness response system) as captured in the BNL illuminated community trends that partners could then work on. To facilitate building the by-name list of Black fathers experiencing homelessness, an intake form was developed and made available on one of our social service partners’ websites. The form collected personal information, homelessness information, parenting status, and support resources needed.

### Referral and linkage system

A key feature of RUSH’s implementation and sustainability was Unite Georgia, a real-time referral and tracking platform supporting coordinated service linkage, which worked well with a shared by-name list of fathers. We collaborated with our partners to support the identification of fathers in need of housing assistance, screening, and referring them to the appropriate resources through the Unite Georgia Network. Two of our medical care partners and two of our social services partners were already part of the Unite Georgia network, and two fatherhood partner organizations joined the network, allowing us to begin testing the process and the Functional Zero approach.

The Unite Georgia system has a consent form for participants, which allowed us to share information and make referrals across partners. We collaboratively developed a flyer for our partners to share in case fathers wanted to reach out to us directly. Due to the lengthy processes and procedures involved in integrating a new system into the public health system, and our abbreviated timeframe for the pilot, we received referrals directly from our public health partner staff or fathers who saw the flyer in their office and entered the referrals into the Unite Georgia system.

### Father interviews

We collaborated with the RPC to develop a semi-structured interview guide for interviewing fathers participating in these services. The interview guide consisted of questions about the fathers, their experiences seeking help, the time spent seeking housing, housing barriers, separation from their children, resources that have been helpful to them, and reasons fathers avoid seeking assistance (See Supplementary File). All fathers who completed an intake form to participate in the project were invited to participate in an interview. Fathers received $50 electronic Visa gift cards for their time. Interviews were audio-recorded and transcribed. The rapid qualitative data analysis process described above was also used to analyze the interview data and interpret results with the RPC.

### Coalition survey

Finally, to assess the feasibility and implementation of the system alignment approach from our coalition members’ perspectives, we administered a confidential online survey via Qualtrics. The 17-item survey included Likert scale and open-ended responses to assess satisfaction with the system alignment approach and coalition participation, recommendations for improvement, and potential for sustainability (See Supplementary File). The survey was emailed to all RPC members. Descriptive statistics were used to analyze the survey data and content analysis used to analyze the open-ended responses.

This project was deemed non-research by the Institutional Review Board in May 2023. We followed verbal informed consent procedures with all interview participants and online consent with survey participants.

## Results

### Exploration phase

#### Homelessness management information system data

Data from the HMIS indicated that 15% of active clients in the HMIS were fathers with children (*n* = 383). Regarding the types of services that men participated in, the majority were referred for various services or entered the coordinated entry process, as Table 1 shows.

**Table 1:** Types of services that fathers experiencing homelessness received

Service	Count	Percent
Services Only	118	30.81%
Coordinated Entry	106	27.68%
Homelessness Prevention	43	11.23%
Rapid Re-Housing	42	10.97%
Permanent Supportive Housing (disability required for entry)	34	8.88%
Housing with Services (no disability required for entry)	20	5.22%
Emergency Shelter	18	4.70%
Street Outreach	2	0.52%
**Grand Total**	**383**	**100.00%**

Source: Atlanta Homelessness Management Information System, 2022

Of the 383 fathers who were in the HMIS in 2022, most were single fathers, not having a partner (53%), and 47% were partnered. Most of these fathers identified as African American/Black/African (93%). Table 2 presents characteristics of mostly Black, single fathers who were in the HMIS in 2023 (*n* = 105).

**Table 2:** Characteristics of single fathers in the atlanta homelessness management information system

Characteristics	Count	Percent
Race		
• Black/African American	98	93.33%
• Multi-Racial	6	5.71%
• Native Hawaiian/Pacific Islander	1	0.96%
Ethnicity		
• Hispanic/Latino	5	4.76%
• Non-Hispanic/Latino	100	95.24%
Veteran Status		
• No	91	86.67%
• Yes	14	13.33%
Disability Status		
• No	57	54.29%
• Yes	45	42.86%
• Data Not Collected	3	2.86%
Total	105	100.00%

Source: Atlanta Homelessness Management Information System, 2023

### Partner interviews

We conducted eight interviews with medical, social services, and public health partners to better understand their readiness, which included assessing processes and systems for identifying, assessing, referring fathers (or those experiencing homelessness), and monitoring data and outcomes. The common themes across these interviews were a lack of data collected specifically about fathers, a lack of intention around serving and documenting fathers, and a need for a comprehensive system and dedicated staff to focus on this population. Strengths included medical care partners’ policies to screen for social determinants of health, which included housing. Another strength was partners’ willingness to address the issue together, even if working with fathers was not their norm, and they acknowledged the inequities in access to resources, specifically for Black fathers. The interviews clarified terminology differences across systems, examined policies for alignment or intervention, and showed partners’ commitment to each other’s goals.

### Implementation

#### Interviews with Black/African American fathers

Among our partners, from March 2024 to May 2024, we identified 14 Black fathers experiencing homelessness in Atlanta. Nine of the fathers met the participant criteria. We piloted this system, using the by-name-list to connect and track these nine identified fathers to housing and other partner resources, and interviewed six fathers.

These fathers had between 1 and 3 children and were without housing. For more details on the fathers, see Table 3. We asked about their experience seeking housing assistance. These fathers had been seeking housing assistance for two to ten years. Three of the fathers we interviewed were negatively impacted by the legal system, which was a barrier to accessing housing services.

**Table 3:** Characteristics of black fathers who were experiencing homelessness and referred to our study

	Age	Children	Parenting Status	Marital Status	Location	Legal System Impacted
Interviewed						
Father 1	28	1	Separated from the child	Single/Never Married (SNM)	College Park	No
Father 2	47	2	Primary Parent	SNM	Decatur	Yes
Father 3	36	2	Separated from children due to homelessness	SNM	Atlanta	Yes
Father 4	33	2	Parenting, but nowhere to stay with children	SNM	Atlanta	Yes
Father 5	46	1	Full Custody	Divorced	Atlanta	No
Father 6	22	1	Co-parenting	SNM	Atlanta	No
Not Interviewed						
Father 7	29	2	Visitation w/children	Undisclosed	Atlanta	Unknown
Father 8	30	3	Co-parenting	Domestic Partner	East Point	Unknown
Father 9	26	Unknown	Separated from children due to homelessness	SNM	Atlanta	Yes

Source: Unite Georgia Participant Intake Data, 2024

Three of the fathers were separated from the family. One father shared that he:

*Had to drop the children off to their grandmother’s home when she’s available so they could have a roof over their head. [I’m] able to assist children in preparing for school*,* take them to school*,* and pick them up from school. This has been the situation for the past 8 months.*

Some of the fathers were unfamiliar with Atlanta’s coordinated entry system, so we referred them to ensure they had full access to those housing resources first. This also helped identify strengths and weaknesses in the current response system. Two fathers had previously gone through coordinated entry and were referred to housing services, but their housing needs remained unmet. One of these fathers received a housing voucher through a rapid rehousing program, but the funding for that program eventually ran out. The fathers shared that there are very few helpful resources available for them and their families. They noted that housing is the primary need, and without it being met, it’s hard to say they have been helped in other ways. Fathers were also asked, besides housing, which services were hardest to obtain, and they mentioned therapy, food, school assistance, medicine, clothing, diapers, and wipes for their children.

We asked fathers why they might not seek assistance, and they shared that they often feel rejected and hopeless. One father shared:

*You don’t think you are ever going to get help*,* you sometimes come in contact with a couple of bad agencies that don’t do too much for you*,* [which] discourages you. It’s hard for men to believe that there is assistance for males*,* and they don’t believe the system is going to work for them. If you don’t fall into the categories of being mentally ill*,* veteran*,* drug addict*,* dysfunctional… as a male*,* it doesn’t seem like there is hope for you.*

Other reasons for not seeking assistance included pride, services not being father-friendly or father-specific, and misperceptions of fathers. One father shared:

*Fathers*,* whenever they’re going through pain and adversities*,* they are not going to walk in smiling but with a passionate approach*,* and they are looked at differently. Passion can be perceived as anger.*

### Sustainment

#### Coalition survey

We had four fathers from community-based organizations and four partners (such as medical, social services, or public health) of the RPC complete the survey. The respondents indicated that the UNITE Georgia system is promising and better than not having a system at all or just relying on phone calls and resource lists. Those who were previously part of the Unite Georgia network had not been using it regularly. All respondents agreed that the project would be successful and supported continuing to test this approach.

*R.U.S.H …is the only [system] of its kind that is tailored towards active fathers. A system that will help Black fathers have the courage to ask for help. (RPC father)*

*As a healthcare partner*,* I look forward to identifying/testing novel methods to identify fathers experiencing homelessness in our healthcare facilities*,* and once identified*,* having effective resources with which the client can be connected. (RPC medical care partner)*

## Discussion

This study contributes to a limited but growing body of research documenting the experiences of fathers, specifically Black fathers, within homelessness response systems. HMIS data indicated that fathers with children comprised 15% of active clients in Atlanta’s system, the majority of whom were Black and unpartnered. This finding aligns with national and international research demonstrating that fathers are frequently under-identified within homelessness data systems due to gendered assumptions about caregiving and family structure, resulting in their systematic invisibility in policy and practice [7, 29, 30]. The issue is therefore not the absence of fathers experiencing homelessness, but rather the absence of intentional identification, documentation, and services designed to support them.

Service utilization patterns further reflect these structural gaps. Most fathers were engaged through referrals or coordinated entry, while relatively few accessed permanent supportive housing or housing with services. This is consistent with prior research showing that family housing interventions are often implicitly designed for mothers and children, while fathers face eligibility barriers related to custody status, shelter policies, or disability requirements [30–32]. The high proportion of fathers without documented disabilities limited access to permanent supportive housing, reinforcing critiques that disability-based eligibility criteria exclude families whose primary needs are housing affordability, economic stability, and protection from structural discrimination rather than clinical diagnoses [33, 34].

The racial composition of fathers in the HMIS, over 90% Black, reflects longstanding racial inequities in housing access, labor markets, and exposure to the criminal legal system. Structural racism has been shown to increase Black men’s risk of homelessness through discriminatory housing practices, disproportionate incarceration, and exclusion from employment and social safety nets [35–37]. In this study, fathers’ reports of legal system involvement as a barrier to housing are consistent with evidence that criminal records significantly reduce access to housing and prolong homelessness, particularly for Black men [38–40].

Partner interviews revealed a lack of father-specific data, intentional outreach, and dedicated staffing to address fathers’ housing needs, which is consistent with research indicating that homelessness, health, and social service systems rarely conceptualize men as caregivers [41, 42]. Despite these gaps, partners demonstrated readiness for change by leveraging existing social determinants of health screening processes to identify men experiencing homelessness and determine parental status. This approach aligns with evidence positioning healthcare systems as critical entry points for identifying housing instability and connecting families to resources [43].

Importantly, partners’ willingness to revise terminology, align policies, and share accountability reflects key elements of effective cross-sector systems change documented in the literature [44]. Their engagement through the RPC illustrates how collaborative governance structures can support equitable service redesign and challenge normative assumptions about who qualifies as a “family” within homelessness systems [45].

Interviews with Black fathers experiencing homelessness underscored the lived consequences of system misalignment. Fathers described prolonged housing instability, often spanning multiple years, which aligns with national evidence showing that men, particularly men of color, experience longer episodes of homelessness and face greater barriers to housing resolution [46, 47]. Several fathers reported family separation due to housing instability, supporting prior research linking homelessness to family fragmentation, adverse child outcomes, and compromised parental well-being [48, 49].

Fathers’ narratives of rejection, stigma, and disengagement from services echo previous research documenting how masculinity norms, racial bias, and unwelcoming service environments deter men from seeking assistance [50]. Participants’ observations that emotional expression or “passion” is often misinterpreted as anger reflect racialized stereotypes that shape provider perceptions and service interactions with Black men, further exacerbating distrust and disengagement [51].

Applying a Functional Zero system-alignment approach within the EPIS framework suggested that meaningful progress is feasible within existing infrastructure. Fathers’ experiences highlighted the precise gaps that Functional Zero models are designed to address, including fragmented data, a lack of real-time tracking, inconsistent referrals, and inequitable access to housing solutions [18, 19, 52]. By improving data visibility through by-name lists and coordinated referrals, the pilot surfaced fathers who were previously hidden and enabled more intentional, equity-focused problem solving across agencies.

Sustainment findings suggest strong potential for institutionalization and scale-up. Coalition members’ support for continuing the approach aligns with evidence that system-level reforms are more likely to endure when stakeholders perceive clear value, feasibility, and alignment with their missions [53]. Expanding Housing First models tailored to father-led families may be particularly impactful, as Housing First has consistently demonstrated improved housing stability and health outcomes without preconditions such as sobriety or service compliance [54, 55]. However, adapting these models for fathers will require policy reforms that recognize diverse family structures and eliminate exclusionary shelter and eligibility criteria.

Long-term success will also depend on collecting and monitoring equity-focused, disaggregated data to assess how race, gender, and parental status shape service access and outcomes [56, 57]. Such monitoring is essential to ensure that system alignment efforts do not inadvertently reproduce existing inequities. Notably, partners’ confidence in the Functional Zero approach indicates readiness for broader implementation and highlights the potential of system alignment strategies to address homelessness among populations that have historically been overlooked.

## Conclusion

Homelessness among fathers presents a complex public health issue with wide-ranging implications for both individual and family health. The EPIS framework offers a robust structure for addressing homelessness among fathers as a multifaceted public health issue, one that necessitates thoughtful examination, strategic planning, evidence-based implementation, and sustainable integration across systems. Taken together, these findings suggest that a Functional Zero approach could be feasible and appropriate for addressing homelessness among Black fathers, provided that structural barriers, particularly those related to identification, documentation, and service exclusion, are discussed explicitly. The strong engagement of partners and positive perceptions of the system alignment strategy demonstrate a high level of readiness for broader implementation and future testing in larger or comparative studies. By centering racial equity, lived experience, and systems coordination, this study offers a promising model for advancing racial and gender justice in homelessness response.

## Supplementary Information

Supplementary Material 1.

## Data Availability

The datasets used and/or analyzed during the current study are available from the corresponding author on reasonable request.
