# Supplementary material for: Developing and testing a system alignment approach to address homelessness among black fathers in Atlanta, GA
Source: BMC Public Health. 2026 Feb 27;26:1098. doi: 10.1186/s12889-026-26791-w (PMC13049797; doi:10.1186/s12889-026-26791-w)
Supplement: Supplementary file 1 — Supplementary Material 1. [file 12889_2026_26791_MOESM1_ESM.pdf]

INTERVIEW GUIDE FOR PARTNERS

Questions

1. (For medical care and public health partners) Is homelessness or housing issues a priority for your organization?
2. What are the challenges to addressing the issue of homelessness in Atlanta?
3. What has contributed to any positive outcomes or successes in addressing homelessness in Atlanta?
4. To what extent are staff aware of the needs and preferences of the fathers being served by your organization?
5. To what extent are staff aware of the barriers of the fathers served by your organization face to accessing housing services/programs?
6. Do you or have you worked with fathers experiencing homelessness?
7. Share the successes (what works) and challenges of working with fathers experiencing homelessness. How do you address the challenges? How do you sustain the successes?
8. What partners do you currently work with to address homelessness or housing issues?
9. What partners do you need or wish you could work with more to address homelessness?
10. What resources would you need to align systems or coordinate with other partners to address housing/homelessness issues among the fathers that you serve?
11. Describe your intake process to identify/assess housing/homelessness issues.
12. What are the common reasons people express as the cause of homelessness/housing challenges?
13. Describe your referral process to address housing/homelessness.
14. How are referrals tracked?
15. How are referrals prioritized?
16. What data systems do you use for intake and referrals?
17. Do you track outcomes for clients/patients? If yes, how?
18. Have you elicited information from clients/patients, specifically fathers, regarding their experiences with the housing program or homelessness response?
19. Have you heard stories about the experiences of fathers with the program or response?
20. Do you collect or have data on the numbers of fathers served, by race?

INTERVIEW GUIDE FOR FATHERS

Questions

- How do you find out about housing services or other services to support you and your family?
- Before you needed housing, did you turn to any organization or person for help? Describe that experience (positive and/or negative)
- How long have you been looking for housing?
- What are the biggest issues that you face when it comes to housing?
- Have you, as a father, ever had to be separated from your family/children for the family/children to be housed? If so, how long were you separated from them?
- Are you familiar with the coordinated entry process for housing? If so, were you able to complete a coordinated entry assessment? Please add any feedback regarding your experience with coordinated entry.
- What types of services would best support you? Your family?
- What services are the most difficult to get?
- Are the services that you have been referred to helpful?
- What are the social service groups, support centers, and meetings that are the most helpful? Why? Least helpful? Why?
- Are there reasons why some fathers might avoid seeking assistance through these types of agencies?
- What would you need to stay in housing long-term, if anything?
- What do you think is the most important issue or issues for systems and services to deal with right now to better serve those seeking housing?
- Are there any questions that I have failed to ask which you would like to raise?

RUSH Coalition Survey

Start of Block: Default Question Block

Q1 Thank you for your time and contributions as a coalition member for our Robert Wood Johnson Foundation-funded project, RUSH, to address homelessness among Black fathers in Atlanta. Please respond to the questions below to share your experiences as a coalition member and feedback on the project and referral system, if applicable. Please share your honest feedback so that we can improve and have a positive impact on your experience, services and individuals served.

Page Break

Q2 Coalition Member Type

- ○ Community Member (honorarium received) (1)
- ○ Organization/Agency Member (2)

Q3 How many meetings did you attend?

- ○ One (1)
- ○ 2-3 (2)
- ○ 4 (3)
- ○ All (4)

Q4 Please share your level of satisfaction with each aspect described below.

	Extremely dissatisfied (1)	Somewhat dissatisfied (2)	Somewhat satisfied (3)	Extremely satisfied (4)
Meeting Content (1)	○	○	○	○
Meeting Frequency (2)	○	○	○	○
Meeting Duration (3)	○	○	○	○
Meeting Time (4)	○	○	○	○

Page Break

Q5 Please share your level of agreement with the statements below.

	Strongly Disagree (1)	Disagree (2)	Agree (3)	Strongly Agree (4)
This coalition was a good use of my time. (1)	○	○	○	○
My knowledge and abilities were used effectively. (2)	○	○	○	○
I am satisfied with the follow through on project activities. (3)	○	○	○	○
I believe this project will be successful. (4)	○	○	○	○

Q6 What do you think worked well with coalition meetings and project activities?

____

____

____

____

____

Q7 How could coalition meetings and/or project activities improve?

____

____

____

____

____

Q8 Is our organization in the Unite GA network?

- No (1)
- Maybe (2)
- Yes (3)

Display this question:

If Is our organization in the Unite GA network? = No

Q9 Would you like to join the Unite GA network?

- No (1)
- Maybe (2)
- Yes (3)

Display this question:

If Would you like to join the Unite GA network? = No

Q10 Why not?

____

Display this question:

If Would you like to join the Unite GA network? = Yes

Or Would you like to join the Unite GA network? = Maybe

Q11 Do you need financial assistance to join the network? (Free to 501c3s)

- No (1)
- Maybe (2)
- Yes (3)

Display this question:

If Is our organization in the Unite GA network? = Yes

Q12 Type of Organization

- Medical Care (1)
- Social Services (2)
- Public Health (3)

Display this question:

If Is our organization in the Unite GA network? = Yes

Q13 How long has your organization been in the Unite GA network?

- Less than 6 months (1)
- Less than one year (2)
- One year (3)
- Two years (4)
- More than 2 years (5)

Display this question:

If Is our organization in the Unite GA network? = Yes

Q14 Have you (or designee) sent referrals?

- No (1)
- Yes (2)

Display this question:

If Have you (or designee) sent referrals? = Yes

Q15 About how many referrals do you send in a week?

____

Display this question:

If Have you (or designee) sent referrals? = Yes

Q16 On average, how many minutes does it take you to enter information and send a referral?

____

Display this question:

If Is our organization in the Unite GA network? = Yes

Q17 Please share your level of agreement with the statements below.

	Strongly disagree (1)	Somewhat disagree (2)	Not Sure (3)	Somewhat agree (4)	Strongly agree (5)
The Unite GA Network referral system is user-friendly. (1)	○	○	○	○	○
The Unite GA Network referral system works better than our previous referral process (e.g., phone, paper forms, etc.). (2)	○	○	○	○	○
I am satisfied with the referral response time. (3)	○	○	○	○	○
I am satisfied with the results of my referrals. (4)	○	○	○	○	○
I received positive feedback from clients/patients about referrals made through the Unite GA Network referral system. (5)	○	○	○	○	○
The Unite GA Network referral system contributes to aligning systems to improve services. (6)	○	○	○	○	○

Q18 Please share any additional comments or feedback on the RUSH project and systems to address homelessness among Black fathers.

____

____

____

____

____

End of Block: Default Question Block
